# Submarine canyons represent an essential habitat network for krill hotspots in a Large Marine Ecosystem

**DOI:** 10.1038/s41598-018-25742-9

**Published:** 2018-05-15

**Authors:** Jarrod A. Santora, Ramona Zeno, Jeffrey G. Dorman, William J. Sydeman

**Affiliations:** 1 Department of Applied Math and Statistics, Center for Stock Assessment Research, University of California Santa Cruz, 1156 High Street, Santa Cruz, CA 95060 USA; 2 Farallon Institute, 101H Street, Suite Q, Petaluma, CA 94952 USA

## Abstract

Submarine canyon systems are ubiquitous features of marine ecosystems, known to support high levels of biodiversity. Canyons may be important to benthic-pelagic ecosystem coupling, but their role in concentrating plankton and structuring pelagic communities is not well known. We hypothesize that at the scale of a large marine ecosystem, canyons provide a critical habitat network, which maintain energy flow and trophic interactions. We evaluate canyon characteristics relative to the distribution and abundance of krill, critically important prey in the California Current Ecosystem. Using a geological database, we conducted a census of canyon locations, evaluated their dimensions, and quantified functional relationships with krill hotspots (i.e., sites of persistently elevated abundance) derived from hydro-acoustic surveys. We found that 76% of krill hotspots occurred within and adjacent to canyons. Most krill hotspots were associated with large shelf-incising canyons. Krill hotspots and canyon dimensions displayed similar coherence as a function of latitude and indicate a potential regional habitat network. The latitudinal migration of many fish, seabirds and mammals may be enhanced by using this canyon-krill network to maintain foraging opportunities. Biogeographic assessments and predictions of krill and krill-predator distributions under climate change may be improved by accounting for canyons in habitat models.

## Introduction

Shallow water topographies, (e.g., sea-mounts) and other bathymetric discontinuities such as submarine canyons are regarded as critical marine habitats, supporting high levels of biodiversity^1–3^. Bathymetric discontinuities may also play an important role in promoting meta-communities^4^, that is, variable interacting predator and prey communities throughout large marine ecosystems. By disrupting hydrodynamics, bathymetric discontinuities may influence the amplitude and direction of ocean currents, which in turn relate to variation in localized upwelling and the development of surface and near-surface structures, such as fronts and eddies^5^. In coastal upwelling ecosystems, submarine canyons are also thought to be important conduits for the transport of deep, nutrient rich waters onto continental shelf waters^5,7–10^. Canyons may also serve as refugia for fish and wildlife during periods of poor ocean productivity^11–13^.

Increased front and eddy formation within and adjacent to canyons^7^ may enhance both the production and retention of plankton and other marine life. To date, coherence in the observed aggregations of zooplankton, forage fish, squid, and top predators (e.g., seabirds and marine mammals) suggest that the location and dimensions of canyons may account for a significant amount of mesoscale variability in the distribution of epipelagic organisms^14–16^. Yet, despite these observations submarine canyons are often neglected in habitat models for mid-water and air-breathing organisms, which usually incorporate simple metrics of bathymetry (e.g., depth, slope and distance to the coast or isobaths^17^). Recent information, derived from satellite observations and digital elevation models provides a valuable new resource^18,19^ for characterizing and studying the importance of submarine canyons on ecosystems at the scale of large marine ecosystems (LMEs). These observations include geospatial information on the dimensions of canyons (e.g., area, length and width), their classification by type (e.g., blind or continental shelf incising), and thus provide a more comprehensive perspective on bathymetric habitat quality throughout ecosystems.

Vertically migrating mesopelagic organisms may be particularly affected by the distribution and characteristics of submarine canyons^20^. In particular, euphausiid crustaceans (hereafter “krill”) play a critical role in the trophodynamics of seabirds, marine mammals and commercially important fish and squid species in many ecosystems worldwide^21,22^ and occur in patchy, yet dense aggregations^23^. Indeed, some of the earliest acoustic surveys of krill aggregations indicated co-occurrence with submarine canyons^14^. The location of krill “hotspots” (i.e., sites of persistently elevated abundance^24^) are regularly observed coinciding with the shelf-break/slope in proximity and within submarine canyon habitat^22,25,26^. Krill are a critical food web component within the California Current Ecosystem (CCE) and sustain wildlife and many commercially important fisheries (e.g., Pacific hake *Merluccius productus*, market squid *Doryteuthis opalescens* and salmon^21,27–29^). Due to their importance, the Pacific Fishery Management Council (PFMC), the federal fisheries management authority for the U.S. west coast, has prohibited the development of krill fisheries, and defined “essential krill habitat” as the entire continental shelf/slope of Oregon, Washington, and California^30^. However, the spatial dynamics of the CCE krill meta-population, as it relates to bathymetric features within this broadly defined essential habitat, is poorly known.

We hypothesize that at the scale of this large marine ecosystem, canyons provide a critical habitat network to maintaining energy flow and trophic interactions in the pelagic ecosystem. To test this hypothesis, we model geospatial information on the distribution of submarine canyons^19^ and krill aggregations derived from extensive hydroacoustic surveys^24,31,32^ (Table S1, Figure S1). We investigate three complementary questions: (a) What is the footprint of all submarine canyon habitat in the CCE, and how do canyon dimensions vary by latitude?, (b) Are krill aggregation hotspots and their persistence related to canyon locations?, and (c) How does krill abundance vary in relation to canyon distribution and dimensions? We apply a previously developed hotspot detection method^32,33^ to enumerate krill hotspots and examine their variability relative to canyon characteristics using Generalized Additive Models (GAMs; See Methods). This study is significant as it demonstrates the importance of accounting for submarine canyon habitat in the biogeography of the CCE. We discuss the importance of canyon habitat as a network of key habitats for foraging predators, and as potential refugia for krill and krill predators relative to climate change impacts on the CCE structure and function.

## Results

### CCE canyons and their characteristics

**Figure 1:**
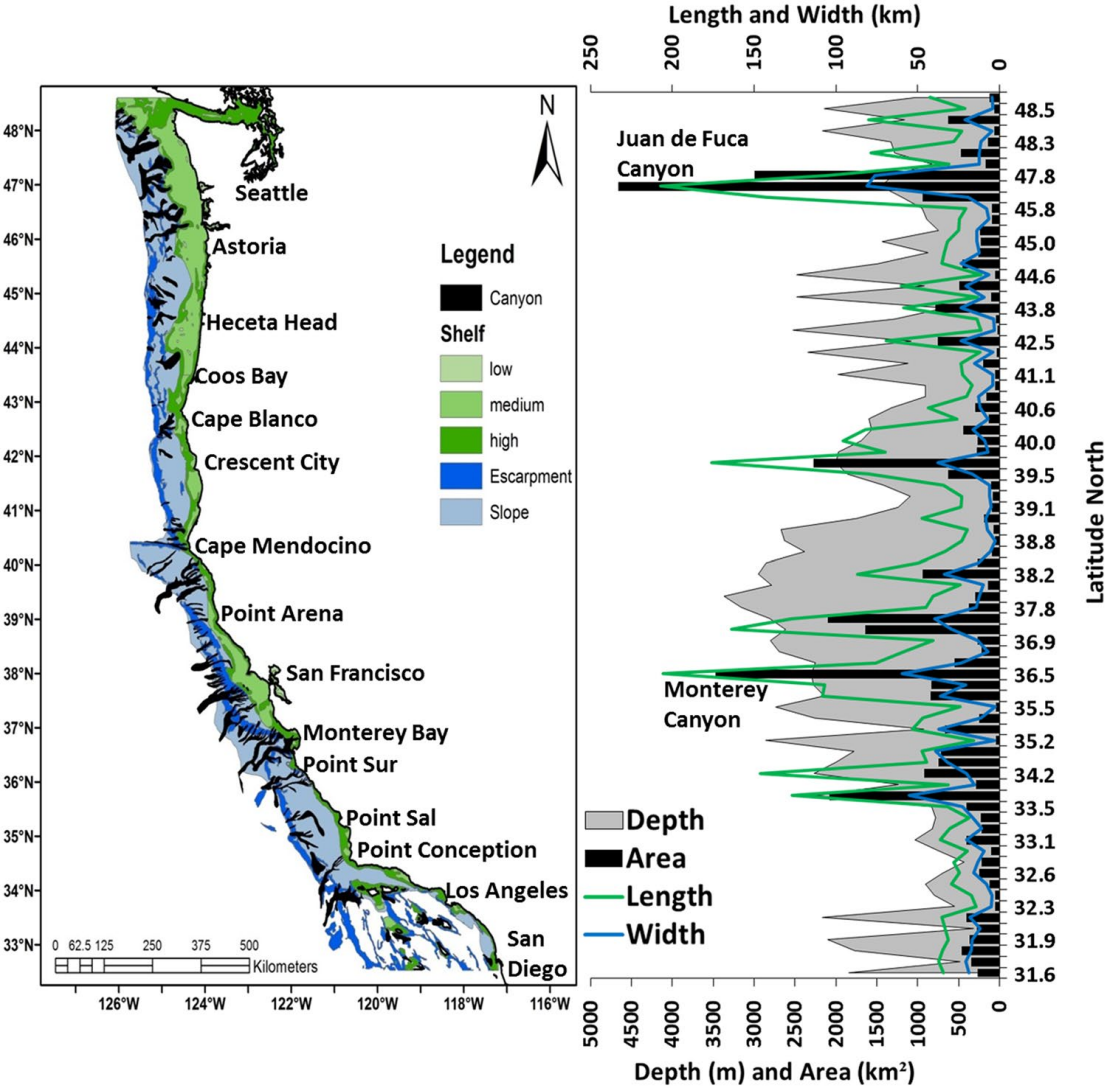
The distribution and dimensions of submarine canyons and classified continental shelf habitat (based on relief) throughout the California Current Large Marine Ecosystem. Map created by the authors using ArcGIS (v 10.3.1; ESRI, 2015); see Methods for description of marine geology features.

**Figure 2:**
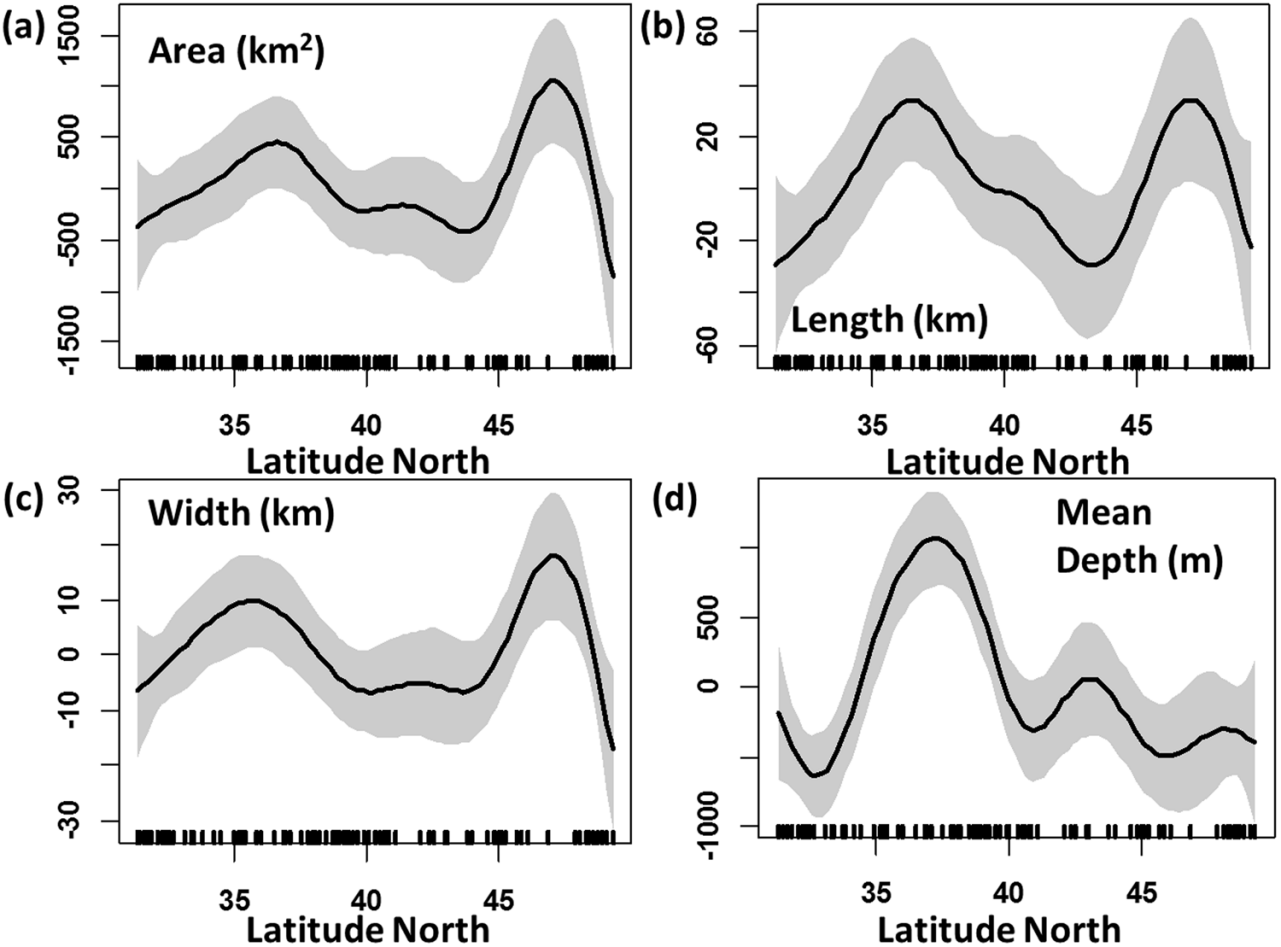
GAM results assessing the latitudinal variability of canyon dimensions across the California Current Ecosystem: (**a**) area (**b**) length, (**c**) width and (**d**) mean depth of canyon. Shaded area indicates 95% confidence limits and tick marks above the x-axis are observations.

There are 80 submarine canyon “systems” (50 blind or non-shelf incising and 30 shelf-incising) located within the CCE (from 31.6°N to 48.5°N). The distribution and geospatial aspects of these canyon systems vary significantly with latitude (Fig. 1). The total area of submarine canyon habitat in the CCE is approximately 41,295 km^2^. The area of individual canyons ranges from 25 km^2^ to 4660 km^2^, and is positively correlated with canyon length and width; 25% are less than 100 km^2^, 66% are between 100–900 km^2^, and 8.7% are greater than 1000 km^2^ (e.g. Monterey and Juan de Fuca Canyons). In comparison to blind canyons, not surprisingly, shelf-incising canyons are significantly larger in area (t = −3.9, p < 0.01), length (t = −4.7, p < 0.01), width (t = −3.6, p < 0.01) and have a greater mean depth (t = 2.8, p < 0.01). There are more canyons concentrated along the central California shelf compared to Oregon and Washington (Fig. 1). All canyon measurements (e.g., width, length, depth) generally increase from south to the north, with a peak off central California around 35–38°N, a decline off Oregon around 40°N, and another peak at latitudes around 45–48°N (Fig. 2). These 2 peaks in canyon dimensions are attributed to the dense clustering of canyon habitat off central California (e.g., Monterey Canyon) and the occurrence of large canyons located offshore of Washington near the Strait of Juan de Fuca (Figs 1–2). The mean depth of canyons tends to be deeper off central California compared to the northern canyon systems off Oregon and Washington (Figs 1–2).

### Relationships between canyons and krill

**Figure 3:**
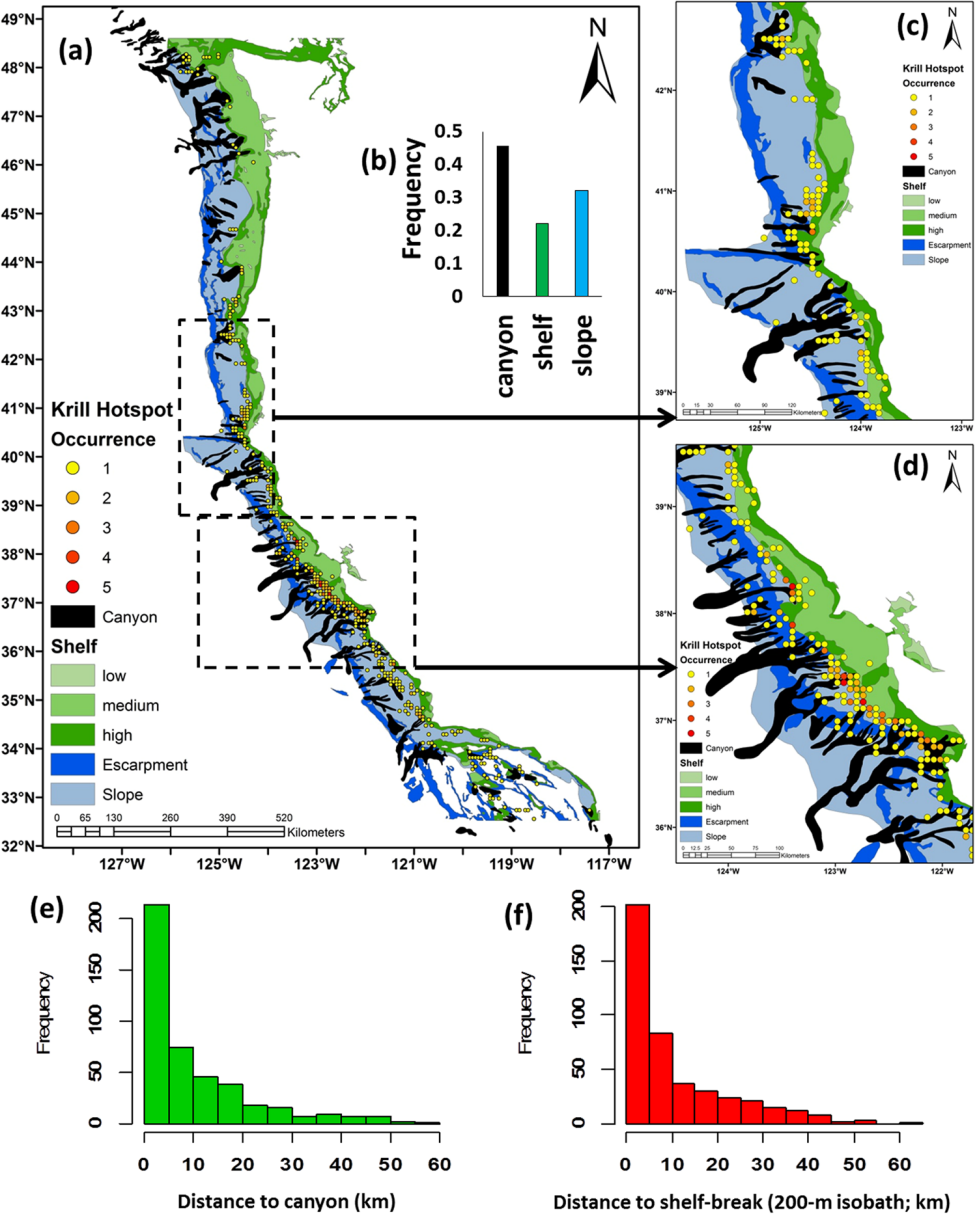
(**a**) Distribution of significant krill hotspots, classified by their re-occurrence, and overlaid on geomorphic features, including: canyon, slope, and shelf habitat; (**b**) inset, highlighting the frequency of krill hotspots detected with geological habitats; (**c**,**d**) areas in the northern and central-northern California Current highlight the association of krill hotspots within canyons and where canyon heads intersect the continental shelf; (**e**,**f**) frequency of krill hotspots relative to distance (km) to nearest submarine canyon and shelf break (200 m isobath contour separating shelf and slope habitats). Map created by the authors using ArcGIS (v 10.3.1; ESRI, 2015); see Methods for description of marine geology features.

Considering all years (2000 to 2015), analysis of Getis-Ord statistics revealed 447 significant krill hotspots; these were mapped to resolve habitat associations and hotspot persistence (Table S.2; Fig. 3a). Linking krill hotspots to canyons indicated 46% of the hotspots occurred within canyons and 30% in adjacent slope habitat (i.e, not intersecting with canyons); therefore, 76% of all krill hotspots in the CCE were associated with canyons (Fig. 3b).

Hotspots were categorized according to their persistence (i.e., number of times a location was a significant hotspot). Off northern and central California, recurring krill hotspots were detected frequently within the canyon regions off Cape Mendocino, and south throughout the dense canyon region of Gulf of the Farallones and Monterey Bay (Fig. 3c,d). Krill hotspots were inversely related to the nearest canyon or the shelf-break region (i.e., 200 m isobath), indicating that the majority of krill hotspots are found within proximity to these features (Fig. 3c,d).

Due to variability of canyon dimensions, krill hotspots were significantly related to sampling effort, canyon area (U = 91.5, z = −4.27, p < 0.0001) and canyon size (U = 109.5, z = −3.93, p < 0.0001; Fig. S2). This is attributed to the fact that shelf-incising canyons tend to be larger and more numerous than blind canyons, and because the survey effort utilized in this study, were more frequently collected off California, where there are more canyons (Fig. 1). This is evident off central California where Monterey Canyon (second largest canyon in CCE; ~12243 km^2^) was highly surveyed and resulted in the highest frequency of krill hotspots (2951 nmi surveyed; hotspots = 43; Figs 3d, S1–2), while Juan de Fuca Canyon in the northern CCE (largest canyon; ~16861 km^2^) was sampled less frequently and fewer hotspots were detected (377 nmi surveyed; hotspots = 3). Therefore, although larger canyons are more likely to contain (and or concentrate) krill hotspots, this finding is somewhat dependent on sampling effort; if large canyons are repeatedly sampled then they should yield more krill hotspot detection.

**Figure 4:**
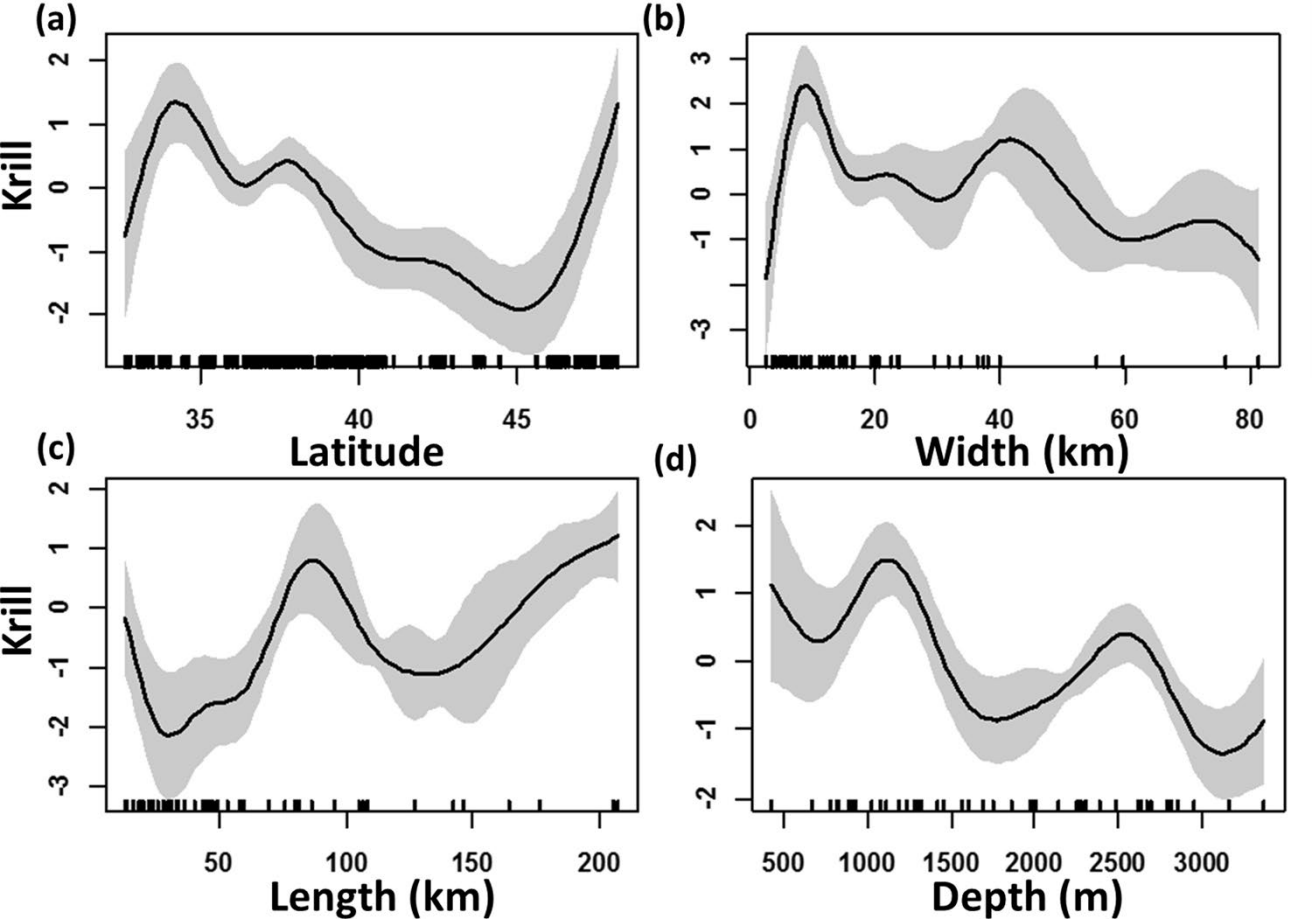
GAM results assessing the (**a**) latitudinal variability of krill relative abundance, and relationship to canyon dimension (**b**) width, (**c**) length and (**d**) mean depth of canyon. Shaded area indicates 95% confidence limits and tick marks above the x-axis are observations.

We used GAMs to model latitudinal variability of krill abundance and association with canyon dimensions throughout the CCE (Fig. 4). Krill varies strongly with latitude, displaying peak abundances off southern (34–35°N) and central (36.5–38°N) California, with a steep decline north of 40°N and throughout Oregon, and a marked increase north of 45°N off Washington (Fig. 4a). This pattern corresponds to the macro-scale pattern of total available canyon habitat throughout the CCE, but also shows there are regions where krill is concentrated without canyons in the vicinity. The GAM applied to krill abundance (observed within canyons) relative to canyon dimensions indicates higher krill abundance associated with canyons widths of 10–20 km and 30–40 km (Fig. 4b) and canyon lengths of 80–100 km and greater than 150 km (Fig. 4c). The functional form of krill abundance and mean canyon depth indicates 2 peaks of higher krill abundance associated with canyons with a mean depth range of 1000–1500 m and 2500 m (Fig. 4d).

## Discussion

In a variety of ecosystems globally (e.g. Antarctic, Bering Sea, California Current), krill concentrations have been found to be associated with the shelf-break/slope region and shallow-water topographies^22,23,25,34,35^. In this study, we sought to refine this understanding of habitat associations in the CCE by testing the hypothesis that within the shelf break/slope region, krill hotspots are found primarily within or in proximity to canyons. We also hypothesized that larger canyons would be associated with more krill hotspots than smaller canyons. Our paper supports both of these hypotheses, and demonstrates the importance of canyon systems and the network of canyons to krill hotspots of the CCE. Many species of fish, birds, and mammals seasonally migrate from the southern portion of the CCE, to the northern portions^29,36–41^ and likely depend on canyon networks for successful feeding and survival. We hypothesize that general pattern of movement for many species of predator in the CCE may be facilitated by the predator’s use of krill hotspots found in association with canyons, and indicate that canyons may have a role in structuring meta-communities in this ecosystem.

### Submarine canyons, krill hotspots and habitat networks

Krill-canyon associations may be explained by currents and structures (e.g., canyon-based fronts and eddies) which retain krill in stable, productive marine microclimates. Previous observational research has shown that krill hotspots are disassociated with centers of active upwelling and Ekman transport in the CCE, suggesting that areas of weaker transport (and/or retention) may explain krill distributions in this ecosystem^24^. Models of krill distribution also indicate that retention is critical to krill hotspot formation and distribution^32,33^; when modeled krill were migrated to sub-surface waters where offshore transport was reduced, modeled distributions matched observations. Both of these approaches, however, do not resolve whether it is actually retentive features (such as fronts or eddies), or lack of offshore advection that is significant to krill distribution. In the present study, we similarly do not resolve the exact mechanism of determining krill distributions, but add the substantial canyon-krill associations, which also clearly suggest that retentive features or lack of advection are important mechanisms for explaining krill distributions in the CCE. The importance of canyons on formation of krill hotspots is likely attributed to the interaction of current flow over steep topography^7,8^. Current flow in the CCE varies seasonally^5,6^ and therefore the formation of krill hotspots may also vary seasonally and spatially. The importance of flow-topography interactions, such a submarine canyon locations, should be explored within ocean-ecosystem models to investigate mechanisms causing the transport and retention of organisms within LMEs.

Whatever the exact mechanism of concentration, the hydrodynamics of submarine canyons appear to concentrate krill and this likely benefits the foraging ecology of planktivorous fish, seabirds, and marine mammals, particularly for highly migratory species, such as sardine, hake, or baleen whales, which move from one region of the CCE to the other in search of food. Given the canyon and krill hotspot associations found throughout the CCE, neighboring canyons that support persistent krill hotspots may be considered a habitat network from the perspective of krill and their predators. While the krill species inhabiting canyons is probably mostly *Euphausia pacifica*^31,42,43^, the community of krill predators using the canyon network undoubtedly varies in time and space, and thereby constitutes a meta-community^4^. From this meta-community (krill-predator) perspective, canyons may represent a foraging network, where encountering food patches (krill concentrations) may be predictable in time and space due to benthic-pelagic coupling mechanisms. Further, mesopelagic and benthic fish species may interact with the pelagic realm through krill concentrations associated with canyons. Therefore, evaluating the connectivity (e.g., size and spacing) and distribution of canyon networks that support krill hotspots and their predators provides a means to effectively characterize the macro-scale heterogeneity and function of LMEs.

### Regional variation in krill Abundance

Our investigation of krill abundance hotspots throughout the CCE largely reflects aggregations of *E*. *pacifica* (i.e., numerically dominant euphausiid and acoustic backscatter target) within submarine canyons throughout the shelf-break zone^13,14,31,43^. However, it is important to note that the acoustics may also detect similar-sized zooplankton and additional survey work (e.g., trawl and net surveys) is needed to determine if the distribution of known krill hotspots relate to other euphausiids and zooplankton. Nevertheless, our assessment of the latitudinal variability of canyon habitat throughout the entire CCE demonstrates the importance of this habitat and suggests how they may influence the mesoscale spatial organization of krill predators. The CCE is also highly organized with upwelling -favorable winds, with key upwelling centers centered on major capes and coastal promontories, which fuel regional nutrient enhancements and variation in ecosystem productivity^44^. In comparison to the northern and southern CCE, the magnitude of upwelling is greatest off northern-central California and this is also the region richest in large shelf-incising canyons (Fig. 1). Therefore, it is not surprising that the highest number and continuous band of krill hotspots are located in this region. In addition to the retentive mechanism discussed earlier, bathymetric-steering of currents and vertical motions within canyons may result in localized upwelling of nutrients that stimulate primary production^5,7^. With the strong regional upwelling, coupled with localized upwelling and the substantial canyon habitat in the region, it is reasonable to conclude that north-central California may be one of the most important and persistent zones of krill productivity and retention in the entire CCE^24,45^. The variation in overall krill biomass across the CCE is not known, but some have argued^46^, that ecosystem productivity is greatest in the northern sector of the CCE off Washington. That may be the case for primary productivity, but given the krill-canyon associations presented here, the high productivity off Washington may not correspond to regional krill biomass. However, to date one of the largest krill aggregations sensed in our studies was found off Nitanet Canyon, Washington, indicating that substantial populations of krill are also found in this region.

### Climate change and ecosystem implications

Global climate change is predicted to impact the structure and function of ecosystems through changes in physical processes (e.g., regional warming, variance in wind-forcing) likely to impact productivity, resulting in pole-ward or equator-ward species distribution shifts^47,48^. However, climate change will not shift the position of bathymetric habitat including canyons, indicating the need to consider the effect of static and dynamic habitats in species distribution modeling^49^. Climate change may influence ocean physics operating within canyons, and that may affect krill distribution and abundance in that manner, but models of species redistributions based solely on isothermal shifts for canyon-dependent species, such as krill, may overestimate distributional shifts. Moreover, during poor ocean conditions (e.g., El Niño), canyons may be considered critical habitats ever more so, as these areas may serve as thermal refugia^50^, where cooler nutrient rich water may be retained, thus supporting higher concentrations of mid-water organisms and their predators during stressful periods^11,12^. Additional research is needed to evaluate this idea more fully.

For aquatic ecosystems, the term “essential habitat” indicates waters and substrate necessary for an organism to feed, grow and reproduce. From a planktonic perspective, essential habitat should also include areas with minimal advective properties that would transport plankton away from preferred habitats. Off the U.S. West Coast, the PFMC broadly defined essential krill habitat as waters from the shoreline extending out to 1,829 m isobath (1000 fathom). Given the apparent patchiness of krill and their tendency to form dense aggregations within and near canyons, this suggests that canyons be considered essential habitat for krill in the CCE. Further, the recurrent concentration of krill hotspots within canyons is likely a fundamental aspect of their life history and suggests canyons are critical for their successful feeding and reproduction^13^^,^^42,43^. For example, the concentration of krill hotspots within canyons may represent a strong connection linking krill population dynamics with regional upwelling of nutrients within canyons, which in turn stimulates primary production^5,6,13^ for krill populations to feed and ensure successful reproduction^43,51^. Conservation and management of upper trophic level predators dependent on krill may be enhanced by emphasis on CCE canyons as sites of potentially high trophic transfer^20,51^. Due to the critical role that krill play in the CCE food web (i.e., principal prey for many predators), the bio-physical coupling of currents, upwelling, retention of nutrients and primary and secondary production within submarine canyons, suggests these locations are potential trophic hotspots that link benthic and pelagic ecosystems^2,20,51^. Already, seamounts are recognized for their role in sustaining high biodiversity and providing critical habitat for many species of conservation concern and fisheries^2^. This study indicates that similar efforts to protect canyon habitat may be warranted, especially since important fisheries (e.g., coastal pelagic species) and endangered species (e.g., blue whale *Balaenoptera musculus*) are likely linked to canyons due to their role in supporting persistent krill hotspots. Furthermore, canyons that cut into shelf habitat appear particularly valuable because they link deep and neritic ocean processes, and are key conduits for supporting coastal krill and dependent predators. Lastly, considering the macro-scale of our synthesis, the relationships between canyons and krill hotspots we found may be extended to other eastern boundary upwelling ecosystems and other systems where canyons and krill are prevalent features. From a global perspective, the importance of krill in marine food webs is clear. Similarly, the coherence between canyon systems and krill hotspots is likely a fundamental aspect of polar and temperate marine ecosystems.

## Methods

### Marine geology

All marine geology features were derived from^19^ and were organized in a geographic information system (GIS) for the CCE (data available from www.bluehabitats.org). We extracted polygons for (a) continental shelf (<200 m) and (b) continental slope (defined as the steepness of the shelf to the continental rise), (c) escarpment (steep sections of the slope) and (d) all submarine canyons between 48.5°N and 32.5°N. Shelf and slope habitats were classified based on water depth; shelf habitat was classified based on low, medium and high relief. Escarpment habitat was identified for steep regions of slope habitat. Along with a unique identification number (based on a global system), all polygons for submarine canyon systems included estimates for canyon area (km^2^), length (km), width (km), and mean bathymetric depth (m;^19^)

### Acoustic surveys

The study domain included data collected from the coast to 150 km offshore and from San Diego, CA (32.5°N) to the ocean entrance of the Strait of Juan de Fuca, WA (48.4°N). Acoustic data were collected during NOAA-National Marine Fisheries Service shipboard surveys during 2000–2015 (n = 29 surveys; Table S.1; Fig. S1). Vessels were equipped with a multi-frequency echosounder (3 transducers 38 kHz, 120 kHz, and 200 kHz) mounted to the hull and operated continuously throughout surveys. A relative krill abundance index was measured as Nautical Acoustic Scattering Coefficient (NASC, m^2^ nmi^−2^), which is a depth-integrated index (integrated to 300 m or shallower) of horizontal krill distribution^52–54^. NASC calculations use a three-frequency delineation method designed to detect euphausiid backscatter^55^. Acoustic echograms were processed using Echoview 4.9 (Myriax Pty Ltd, Hobart, AU) and visually examined to check for interference from other echosounders and bottom echoes. Data were excluded when vessel speed was less than five knots and at stations to avoid noise contamination from net tows or masking from surface bubble clouds. Data were also excluded when there was surface or bottom contamination that could affect integrated NASC^24,31^. Within the survey area, previous research indicates that the acoustic sampling and technique, suggests that NASC reflects the abundance and distribution of adult *Euphausia pacifica*^33^ (i.e., the numerically dominant euphausiid). Due to the heterogeneous distribution and concentration of zooplankton, we acknowledge that the acoustics may also reflect other similarly-sized targets, although krill are likely the dominant backscatter, especially within the shelf-break region. Additional work is needed to verify that the acoustic methods correspond with krill derived from trawl surveys collected throughout the CCE. Approximately 60,000 nmi of survey trackline were sampled to assess krill distribution and abundance, resulting in 8,000 nmi in submarine canyon, 16,000 nmi in slope habitat, and 24,000 nmi in shelf habitat (Fig. S1). See http://www.faralloninstitute.org/data for a description and availability of acoustic surveys.

### Geospatial analysis and modeling

In ArcGIS (v 10.3.1; ESRI, 2015), a grid-based system was used to define the extent of the surveys and to partition grid cells into 25 km^2^ geo-referenced cells^24,32,34^ (Fig. S1); grid cell size was chosen based on a scale-dependent assessment of the anisotropic spatial variability (i.e., directional autocorrelation) of krill abundance^24,32^. Geomorphic features (e.g., slope, shelf, canyon in polygon format) were also linked to the acoustic grid cells. Integrated NASC was merged with grid cells and survey effort was determined by counting total number of 1-nmi samples obtained within each grid cell and the spatial mean abundance of krill per grid cell^34^.

A Getis-Ord hotspot analysis was conducted to analyze the degree of local clustering relative to background krill abundance and spatial variability^56^, independently for each survey^32^. Moran’s I tests for 2-dimensional spatial autocorrelation of NASC values^24,32^ indicated a fixed distance band of 12 km was used to define local areas for this analysis. Each sampled grid cell was assigned a relative Z-score to measure the degree of spatial clustering (i.e., more intense hotspot have higher positive Z-scores). Significance of Z-scores was assessed at p < 0.05 were considered a significant hotspot of krill abundance^32,33^.

Krill hotspots were spatially-linked to canyons if they overlapped with the boundaries, thus allowing the calculation of number of hotspots per individual canyon system. All hotspots that occurred on canyons were assigned to canyon habitat even if it intersected with slope, or escarpment habitats. Slope hotspots were assigned to slope habitat if they occurred on the slope habitat and did not overlap with a canyon. Shelf hotspots were assigned to shelf habitat if they did not overlap with canyon or slope. Frequency of hotspots per canyon, slope and shelf habitats was calculated based on the number of hotspots overlapping with each geomorphic feature relative to the total number of hotspots. Further, to assess the degree of association among krill hotspots, canyons and the shelf-break region, the distance from krill hotspots was measured (km) to the nearest canyon and shelf-break slope region (demarcated by 200 m isobath). Krill hotspots located within a canyon or on the 200 m isobaths were assigned a zero distance. We calculated distance-based histograms to illustrate the coherence among krill hotspots, canyons and the shelf-break.

Generalized additive models (GAMs) were used to assess the variability of canyon dimensions as a function of latitude for the entire CCE, and were selected based on previous analyses^32,34^ and for their ability to identify important non-linearities of geospatial features. The *mgcv* package in the statistical program R was used to calculate GAMs. The GAM for canyons was specified as: Canyon Aspect ~ *s*(Latitude), where Canyon Aspect is either area, length, width, or mean depth per individual canyon system, and *s* is smoothing spline; a separate mode was run for each canyon aspect in order to evaluate the latitudinal variability of canyon habitat over the CCE. GAMs were used to evaluate the latitudinal variability of total krill abundance (for all sampled habitat; derived from^32^) and relative to canyon aspects. The GAM to assess krill abundance (averaged NASC per grid cell) relative to canyon features was specified as: Krill abundance ~ *s*(length) + *s*(width) + *s*(mean depth); area was not included in the model because it is a product of canyon length and width.

### Data Availability

The datasets generated during and/or analysed during the current study are available from the corresponding author on reasonable request.

## Electronic supplementary material

Supplemental material
