# Supplementary material for: Submarine canyons represent an essential habitat network for krill hotspots in a Large Marine Ecosystem
Source: Sci Rep. 2018 May 15;8:7579. doi: 10.1038/s41598-018-25742-9 (PMC5954138; doi:10.1038/s41598-018-25742-9)
Supplement: Supplementary file 1 — Supplemental material [file 41598_2018_25742_MOESM1_ESM.pdf]

Supplemental Material:

Submarine canyons represent an essential habitat network for krill hotspots in a Large Marine Ecosystem

Jarrod A. Santora[1, 2*], Ramona Zeno[2], Jeffrey G. Dorman[2], William J. Sydeman[2]

[1]Department of Applied Math and Statistics, University of California Santa Cruz, Santa Cruz, CA, 95060, USA
[2]Farallon Institute, 101 H Street, Suite Q, Petaluma, CA, 94952, USA
*E-mail: jsantora@ucsc.edu

Table S.1: Hydroacoustic data was collected on Juvenile Rockfish Surveys, YOY Salmon Surveys and Zooplankton Surveys (2000-2006 2008-2015) March through August. (Year, Survey, Nautical Miles sampled, start date of the survey and end date of the survey)

Year/ Survey ID	Nmi sampled	Start date	End Date
**2000/ JRS2000**	1315	20000512	20000609
**2001/ JRS2001**	1344	20010509	20010608
**2002/ JRS2002**	714	20020511	20020602
**2003/ JRS2003**	718	20030513	20030601
**2004/ JRS2004**	1258	20040505	20040610
**2005/ JRS2005**	2215	20050504	20050611
**2006/ar0605**	2736	20060508	20060519
**2006/JRS2006**	740	20060507	20060617
**2008/JRS2008**	3283	20080505	20080618
**2008/m20807**	1433	20080712	20080721
**2009/JRS2009**	1056	20090520	20090625
**2009/m20901**	2208	20090323	20090409
**2009/m20904**	1327	20090811	20090819
**2010/bpa2010**	2176	20100620	20100629
**2010/FR1001**	2088	20100630	20100714
**2010/jrs2010**	700	20100501	20100508
**2010/mf1002**	617	20100524	20100528
**2010/mf1004**	1088	20100803	20100810
**2011/fr0611**	1014	20110619	20110628
**2011/FR1101**	2921	20110630	20110716
**2011/fr1105**	894	20110501	20110509
**2012/jrs2012**	3871	20120507	20120616
**2012/OS1201**	1219	20120611	20120626
**2013/jrs2013**	4203	20130509	20130620
**2013/salmon2013**	1700	20130709	20130726
**2014/JRS2014**	4510	20140502	20140616
**2014/salm2014**	1909	20140629	20140724
**2015/JRS2015**	9728	20150502	20150614
**2015/sal2015**	1126	20150618	20150708

Table S.2: Summary of NASC hotspots occurring within each geomorphic habitat (canyon, shelf). Mean gridded NASC, Maximum NASC, Standard deviation of NASC, Maximum Getis-Ord Gi* z-score, Mean Getis-Ord Gi* z-score, standard deviation of Getis-Ord Gi* z-scores, count of hotspots and frequency (number of hotspots on an individual feature/ total number of hotspots).

Habitat	Mean NASC	Max NASC	SD NASC	Max of Gi* z-score	MEAN Gi* z-score	SD Gi* z-score	Count of hotspots	Frequency of hotspots
**Canyon**	1281.40	20756.03	1209.04	22.10	3.71	2.39	204	0.46
**Shelf**	1072.71	16009.88	1006.22	10.97	3.86	2.00	99	0.22
**Slope**	125.59	7441.24	1160.79	17.39	3.67	2.17	144	0.32

Supplemental Figure S.1

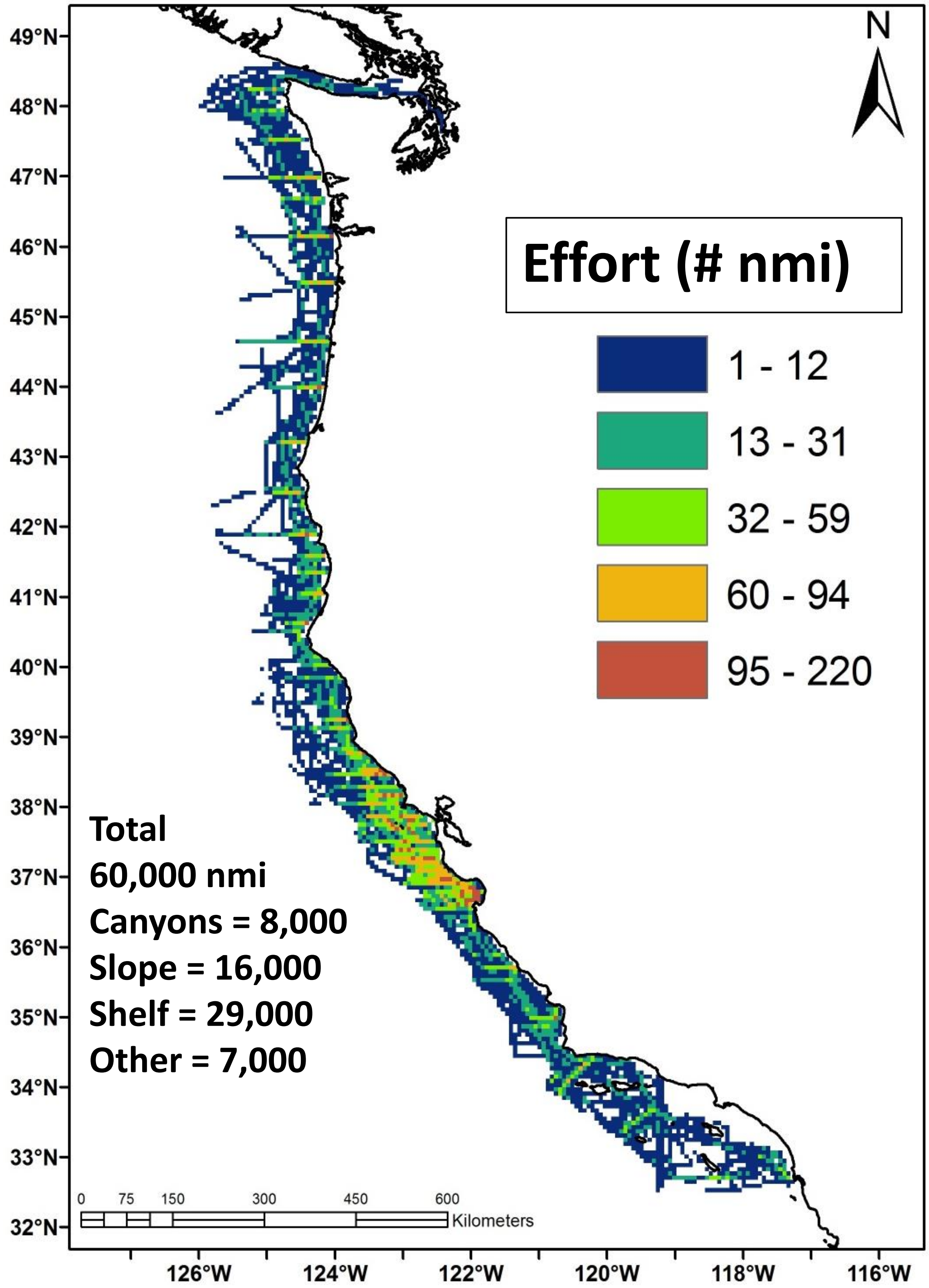

Figure S.1. Coast wide survey effort including all 29 surveys. Data collected 150km offshore from San Diego, CA (32.5°N) to the Strait of Juan de Fuca, WA, at the Canadian border (48.4°N). (Grid averaged sampling effort (each grid cell = 25 km^2). Map created by the authors using ArcGIS (v 10.3.1; ESRI, 2015).

Supplemental Figure S.2

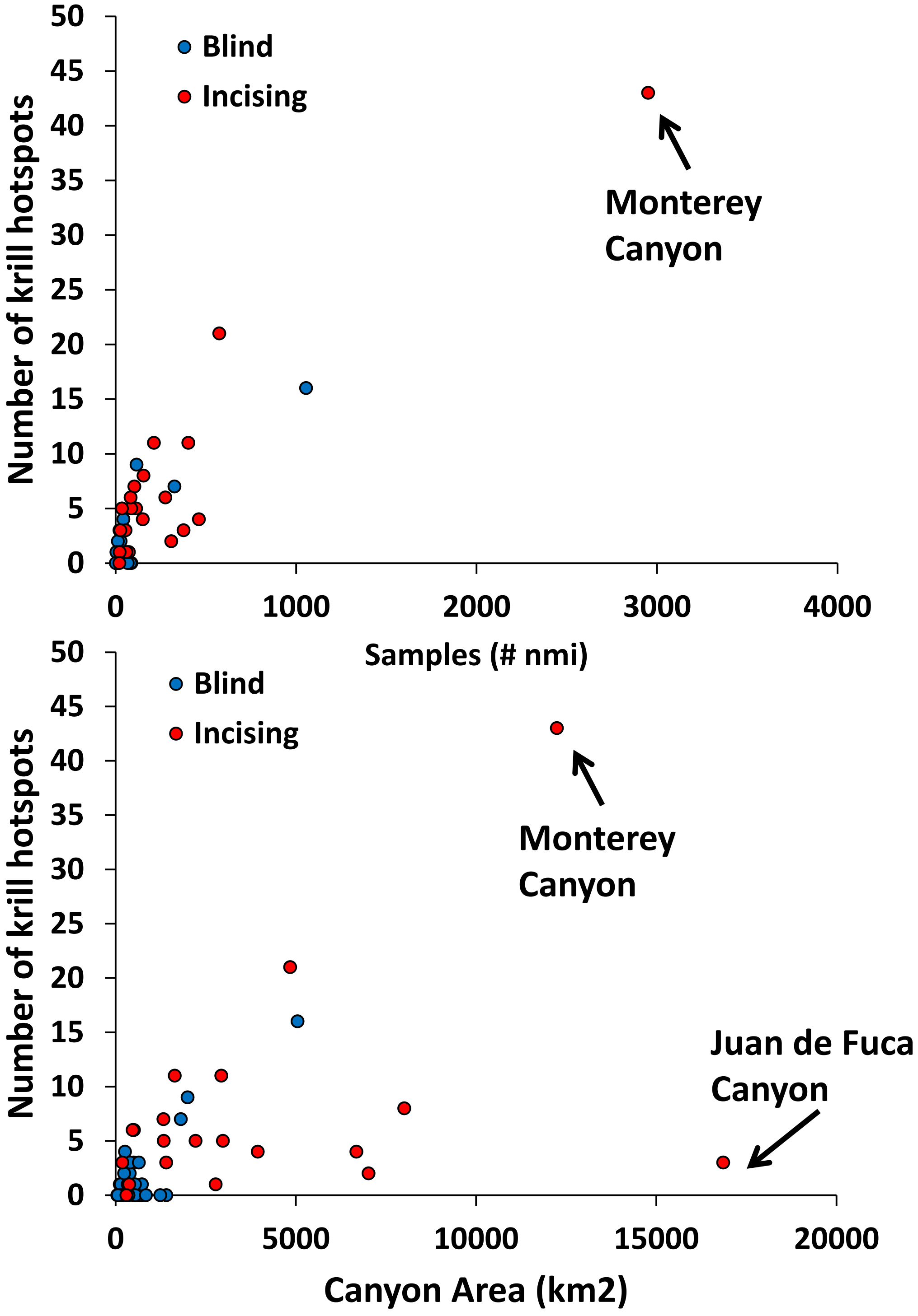

Figure S.2: Relationships between the number of krill hotspots detected with (a) sampling effort and (b) canyon area, according to blind and shelf-incising canyons.
